# Creating scenarios of the impact of copd and their relationship to copd assessment test (CAT™) scores

**DOI:** 10.1186/1471-2466-11-42

**Published:** 2011-08-11

**Authors:** Paul W Jones, Margaret Tabberer, Wen-Hung Chen

**Affiliations:** 1 Division of Clinical Science, St. George's University of London, London, UK; 2 Global Health Outcomes, GlaxoSmithKline, London, UK; 3 Center for Health Outcomes Research, United Biosource Corporation, Bethesda, MD, USA

## Abstract

**Background:**

The COPD Assessment Test (CAT™) is a new short health status measure for routine use. New questionnaires require reference points so that users can understand the scores; descriptive scenarios are one way of doing this. A novel method of creating scenarios is described.

**Methods:**

A Bland and Altman plot showed a consistent relationship between CAT scores and scores obtained with the St George's Respiratory Questionnaire for COPD (SGRQ-C) permitting a direct mapping process between CAT and SGRQ items. The severity associated with each CAT item was calculated using a probabilistic model and expressed in logits (log odds of a patient of given severity affirming that item 50% of the time). Severity estimates for SGRQ-C items in logits were also available, allowing direct comparisons with CAT items. CAT scores were categorised into Low, Medium, High and Very High Impact. SGRQ items of corresponding severity were used to create scenarios associated with each category.

**Results:**

Each CAT category was associated with a scenario comprising 12 to 16 SGRQ-C items. A severity 'ladder' associating CAT scores with exemplar health status effects was also created. Items associated with 'Low' and 'Medium' Impact appeared to be subjectively quite severe in terms of their effect on daily life.

**Conclusions:**

These scenarios provide users of the CAT with a good sense of the health impact associated with different scores. More generally they provide a surprising insight into the severity of the effects of COPD, even in patients with apparently mild-moderate health status impact.

## Background

Understanding a chronic obstructive pulmonary disease (COPD) patient's health status is an integral part of overall patient management. International guidelines on the management of COPD recommend that both lung function and health status are monitored regularly to guide any changes in treatment [1], and both the European Respiratory Society and the American Thoracic Society recommend that health status should be assessed as an outcome in clinical trials of new and existing pharmacological therapies for treatment of COPD [2]. A number of different questionnaires are available that assess health status in COPD, these include the Chronic Respiratory Questionnaire (CRQ) [3], the Clinical COPD Questionnaire (CCQ) [4], the St. Georges Respiratory Questionnaire (SGRQ) [5] and a revised form of the SGRQ, SGRQ-C, which retains the accuracy and responsiveness of the SGRQ but which features fewer questions; scores obtained with the SGRQ and SGRQ-C are directly comparable [6].

All health status questionnaires require reference points so that physicians can attach meaning to their scores. One approach is to calculate a minimum clinically important difference (MCID). This allows users of the questionnaire to distinguish clinically relevant differences within patients, for example in an interventional trial, or in the same patient over time, for example before and after pulmonary rehabilitation. However, the MCID only provides an estimate of the minimum worthwhile difference and does not describe in what nature the health status has changed [7]. Another approach is to relate scores to clinical scenarios. This has been done to illustrate the MCID (4 units) for the SGRQ [8], where the scenarios are based on responses to individual questions. For example, a scenario describing a patient who; *no longer takes a long time to wash or dress, can now walk up stairs without stopping and go out for entertainment *relates to a pattern of change in the patients health status correspondent to a 4-unit improvement. Despite these useful descriptive characteristics, within the field of pulmonary medicine there has been no attempt to create scenarios that can provide clinicians with descriptions that cover the entire range of a health status scale.

We have recently described the development of a new simple health status questionnaire, the COPD Assessment Test (CAT™) [9,10], which correlates very well with the SGRQ-C in stable COPD patients (r = 0.80) and in patients experiencing an exacerbation (r = 0.78). This paper describes the development of descriptive scenarios for the CAT based upon the content of the SGRQ-C.

'Mapping' the contents of SGRQ-C to the CAT was possible as the CAT was developed using Rasch methodology while development of the SGRQ-C involved retrospective Rasch analysis of the original SGRQ to identify items that could be removed. Consequently it has been possible to convert both questionnaires scores to a common unit of measurement that then allows direct comparison between CAT scores and SGRQ item severity scores, and subsequent mapping of SGRQ-C scenarios to the full spectrum of CAT scores.

## Methods

### Comparison of CAT and SGRQ scores

The correlation between SGRQ and CAT scores in stable patients is good (r = 0.80) [9], however a better method of assessing the agreement of two instruments designed to measure the same thing is a technique known most commonly as the Bland and Altman plot [11]. This tests whether the two instruments behave in the same way across the entire scaling range of the instruments, by plotting the difference between measurements made by the two instruments in the same individual against the mean of the two measurements. The differences should be small across the scaling range and have no, or only a very small, correlation with the means. The CAT scale ranges from 0 to 40 while the SGRQ scale ranges from 0 to 100, therefore in order to create a Bland and Altman plot, it was necessary to multiply the CAT score by 2.5 to make the scaling range directly comparable with that of the SGRQ. This CAT score was called the 'adjCAT'.

### Rasch analysis

Rasch methodology is based upon testing the performance of the Guttman scaling properties of a questionnaire's constituent items [12-14]. The key property of this type of scale is the assumption that, for an item of given severity, a patient will have a high probability of responding positively to items that indicate lesser severity than the item in question and a lower probability of responding positively to items that reflect greater severity, when a positive response denotes the presence rather than the absence of an impairment or disability. Rasch modelling was used in the development of CAT, as described elsewhere [9]. Using this approach, severity is calculated as the log odds (logit) of a patient affirming that item 50% of the time. The average severity of the items is conventionally fixed at zero logit, therefore a mild score has a negative logit and a severe score has a positive logit.

### Scoring the CAT

The item reduction stage of CAT development used Rasch analysis to determine the eight items that formed the final questionnaire [9]. This model confirmed that the CAT met the requirements for a unidimensional scale. As a result, a reliable score of overall health status could be calculated using the simple sum of the patient's responses to the items. In a questionnaire developed using Rasch modelling, the relationship between the questionnaire's score, calculated as the simple sum, and severity scored in logits forms a mathematically defined relationship. A conversion table allows CAT scores to be converted to logits or vice versa. An abbreviated version is shown in Table 1 and the full version is included in Additional File 1: Appendix 1.

**Table 1:** Abbreviated conversion table from CAT score to logits

CAT score	CAT logits
0	-4.31

5	-1.90

10	-1.03

15	-0.43

20	0.07

25	0.55

30	1.07

35	1.82

40	4.01

### Scoring the SGRQ

Scores for the SGRQ are calculated by applying empirically derived weights to the patients' responses to each item. This is an entirely different methodology from that used for scoring the CAT and meant that a simple direct mapping exercise to relate CAT scores to SGRQ scores was not possible. However, a recent exercise to refine the SGRQ to produce the SGRQ-C used Rasch methodology [6]. This process also provided estimates of the severity of each item calculated as logits, which made it possible to compare CAT scores and SGRQ items using the same metric. Most of the items in the SGRQ are dichotomous, so we used the logit for that item. About 15% of the items have multiple response categories and in these cases we used the logit for each category of response.

### Mapping CAT scores to SGRQ items

**Figure 1:**
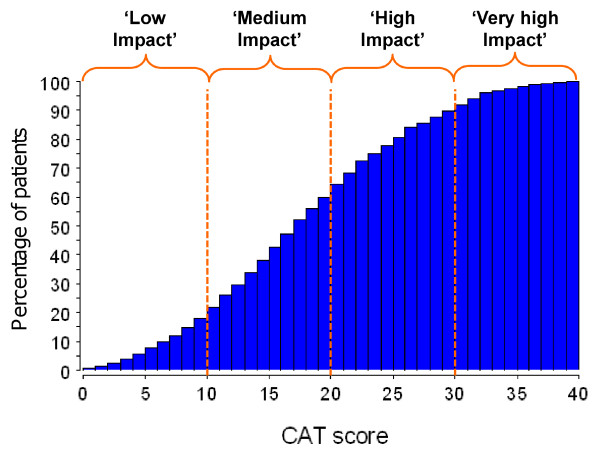
**Cumulative frequency distribution of CAT scores**.

**Figure 2:**
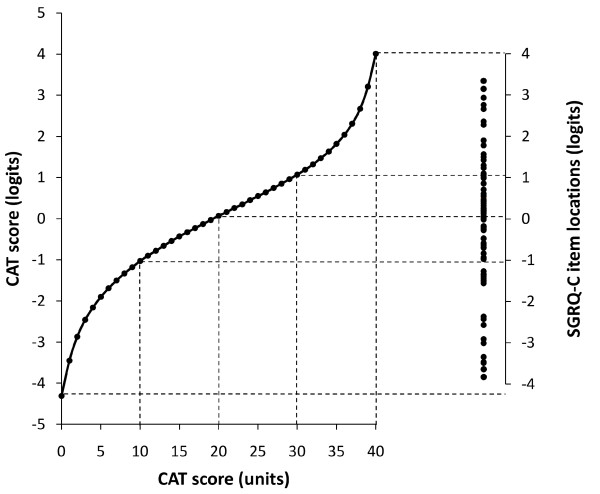
**Mapping CAT scores to SGRQ items**. See text for full explanation.

CAT scores had already been categorised into severity bands, as described in the CAT users guide (http://www.catestonline.org): Low Impact (CAT score 1 to 10), Medium Impact (11 to 20), High Impact (21 to 30), Very High Impact (31 to 40) (Figure 1). This categorisation took place prior to the analysis presented here and was not based on any knowledge of mapped SGRQ items. Scenarios were created for each category by mapping them to SGRQ-C items of corresponding severity using CAT categories and SGRQ-C item severity expressed in logits (Figure 2).

### Patients

Patients were recruited from sites in Belgium, France, Germany, The Netherlands, Spain, UK, and USA. Full details of patient recruitment and questionnaire administration are available elsewhere [9]. The study was conducted in compliance with the Declaration of Helsinki with ethics approval provided by local ethics committees. All patients provided written informed consent prior to study procedures.

## Results

### CAT categories within a COPD population

Full details of these patients have been published elsewhere [9], in brief their mean age was 66 years, 32% were female and their mean FEV_1 _was 58% predicted. In Figure 1, the CAT severity categories are superimposed upon a cumulative frequency distribution of CAT scores in 1503 patients recruited from Belgium, France, Germany, The Netherlands, Spain, UK, and USA. The proportion of scores was 18% Low Impact, 43% Medium Impact, 28% High Impact, and 11% Very High Impact.

### Correlation with SGRQ

**Figure 3:**
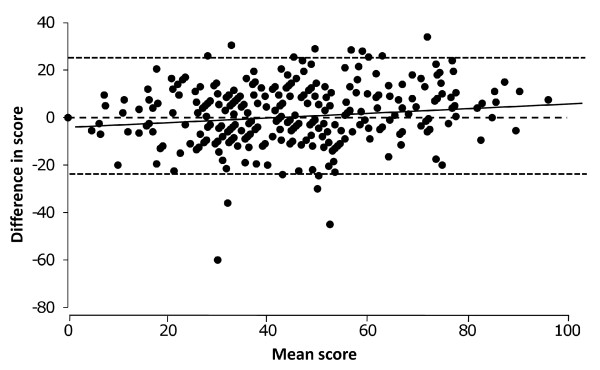
**Bland and Altman plot of SGRQ and adjCAT scores**. CAT scores were converted to 0 to 100% (adjCAT) to match SGRQ scores. The X axis is the mean of the SGRQ and adjCAT scores; the y axis is SGRQ-adjCAT score. The correlation for a linear regression was r = 0.16, p = 0.005.

SGRQ and CAT scores were obtained in the same patients. The Bland and Altman plot in Figure 3 showed a very stable relationship across the scaling range, although there was a very small positive correlation (r = 0.16, p = 0.005). At the mild end of the CAT scale the score slightly over-estimated severity by a small amount (SGRQ = 0, adjCAT = 5, equivalent to 2 CAT units) and at the severe end it slightly under-estimated severity (SGRQ = 100, adjCAT = 92.5, equivalent to 37 CAT units). This level of agreement was sufficient to permit direct mapping between SGRQ and CAT for the purpose of creating these scenarios.

The Bland and Altman plot also shows the limits of agreement between CAT and SGRQ; 31% of the score differences are less than 5 points (i.e. difference of ≤5%) and 60% are less than 10 points (difference of ≤10%), and 90% are less than 20 points (difference of ≤20%). These numbers show substantial agreement between the CAT and SGRQ.

### Creation of CAT scenarios

The SGRQ-C items associated with each of the CAT categories are listed in Table 2. A representative selection of these items was used to create the brief scenarios described in the CAT user guide [http://www.catestonline.org].

**Table 2:** SGRQ-C items grouped by corresponding CAT severity category

Low Impact (CAT 1-10)	Medium Impact (CAT 11-20)	High Impact (CAT 21-30)	Very High Impact (CAT 31-40)
Breathless several days a week **(-3.86)**	Housework takes long or stop for rests **(-0.91)**	Chest causes lot of problems or most important problem **(0.15)**	Cough causes tiredness **(1.13)**

Breathless walking up hills **(-3.72)**	Breathless most days a week **(-0.80)**	3 or more attacks of chest trouble in last year **(0.20)**	Takes a long time to get washed or dressed **(1.24)**

Difficult to carry heavy loads, etc **(-3.16)**	Bring up phlegm several days a week **(-0.77)**	Get afraid/panic when can't get breath **(0.21)**	Breathless walking around home **(1.47)**

Have to stop/slow down if hurry/walk fast **(-2.98)**	Wheezing attacks only with chest infections **(-0.68)**	Breathless walking on level ground outside the house **(0.32)**	Chest trouble is a nuisance to family, friends **(1.49)**

Chest condition causes a few problems **(-2.91)**	Cough several days a week **(-0.64)**	Wheezing attacks several days a week **(0.36)**	Cannot take bath/shower or takes long time **(1.55)**

Difficult to walk up hill, light gardening, etc **(-2.64)**	Wheezing attacks a few days a month **(-0.35)**	Cough and/or breathing embarrassing in public **(0.47)**	Cannot go out for entertainment **(1.87)**

Most days are good in average week **(-2.63)**	1-2 attack of chest trouble in last year **(-0.30)**	Cough and/or breathing disturbs sleep **(0.49)**	Coughs hurts **(2.11)**

Stops 1 or 2 things **(-2.15)**	Bring up phlegm most days a week **(-0.21)**	Feel not in control of chest problem **(0.49)**	Cannot do housework **(2.20)**

Breathless walking up a flight of stairs **(-2.15)**	A few good days in an average week **(-0.19)**	Wheezing attacks most days a week **(0.58)**	Have become frail or invalid because of chest **(2.42)**

Cough only with chest infections **(-2.06)**	Cough most days a week **(-0.09)**	Stops patient doing most things they want to do **(0.60)**	Cannot go out of house for shopping **(2.69)**

Walk slower than others or stop for rests **(-1.52)**	Breathless when bending over **(-0.07)**	Breathless getting washed/dressed **(0.62)**	Stops patient doing everything they want to do **(3.11)**

Breathless only with chest infections **(-1.52)**	Wheeze worse in morning **(-0.02)**	No good days in average week **(0.63)**	Cannot move far from bed or chair **(3.40)**

Get exhausted easily **(-1.47)**		Breathless when talking **(0.81)**	

Walk slowly or stop walking one flight of stairs **(-1.14)**		Exercise felt not to be safe **(0.92)**	

Bring up phlegm only with chest infections **(-1.07)**		Everything seems too much of an effort **(0.92)**	

Usually cannot play sports or games **(-1.05)**			

Figure in bold brackets is the SGRQ-C item's severity expressed in logits; low score (negative) = mild, high score (positive) = severe

### COPD ladder of severity

An alternative method of showing the relationship between CAT score and SGRQ-C scenarios is shown in Table 3. Representative items for each 5-point step along the CAT are listed in ascending order of severity. This is termed a 'ladder of severity' because at each level, it is likely that the patient will also have experienced the development of many of the health affects associated with the milder steps up to their current severity.

**Table 3:** COPD ladder of poor health

CAT score	Descriptions
40	Cannot move far from bed or chair Have become frail or an invalid Cannot do housework

35	Cannot take bath/shower or takes a long time Breathless walking around the home Chest trouble has become a nuisance to friends/relatives

30	Everything seems too much of an effort No good days in the week Stops patient doing most of what they want to do

25	Feel that not in control of chest problem Cough/breathing disturbs sleep Get afraid or panic when cannot get breath

20	Wheeze worse in the morning Breathless on bending over Wheezing attacks on most days

15	Cough several days a week Breathlessness on most days Housework takes a long time or have to take rests

10	Usually cannot play sports or games Gets exhausted easily Walk slower than other people or stop for rests

5	Breathlessness stops patient doing one or two things Chest condition causes a few problems Breathless walking up hills

This ladder is a Guttman scale, so at any given level of CAT score, it is probable that the patient will experience most of the less severe descriptions

## Discussion

This analysis has used an objective scientific method to create clinical scenarios that are associated with different scores obtained with a new measure of impaired health status for COPD. A number of factors made this possible: 1. Rasch-imputed mapping has been used successfully in other diseases to map measures between two instruments [15], and develop scenarios corresponding to outcomes within an instrument [16]; 2. CAT scores and SGRQ-C scores correlate well across the entire scaling range from very mild to very severe; 3. The CAT scores and SGRQ-C items could be expressed in the same units of measurement; 4. The SGRQ is made up of sufficient items (some of which have multiple response options, each with its own calculated logit value) to permit relatively rich descriptions, so each CAT category was associated with 12 or more SGRQ-C items; 5. Rasch models are thought to be sample independent [17], thereby permitting comparisons between different groups of patients.

This approach enabled us to provide scenarios that describe patients exhibiting CAT scores ranging from the very mild to the very severe. For example, patients who become breathless while walking up hills fall into the Low Impact CAT category, while those who become breathless while walking around the home fall into the Very High Impact category. These scenarios allow for a more rounded understanding of the effects of COPD associated with different CAT scores and for a more ready appreciation of what the scores mean for the patient in terms of the effect of COPD on their lives. The data used to map SGRQ-C items to CAT severities were derived from multiple countries and, during the CAT's development, items that performed differently in different countries were excluded. For these reasons, we believe that large regional variation in the scenarios is unlikely and that they are applicable wherever a valid translation of CAT is available (current list available at http://www.catestonline.org).

There are, however, some weaknesses with the approach used here. Ideally, the Rasch analysis would have been performed on the same patient population as that used for the CAT analysis, but this was not possible for resource reasons. However, we have shown previously that within a study population repeat estimates of item severity calculated using Rasch analyses were very stable over time [13]. The items in the SGRQ-C don't provide a fully comprehensive description of every effect that COPD can have on a person, but there are common effects that should be experienced by most patients. Some of the items do not seem intuitively to be of the 'right' severity, for example bringing up phlegm only with chest infections is associated with a similar degree of severity as having to stop when walking up stairs, however these severity estimates were calculated using data from approximately 900 COPD patients [6] so they should be reliable. Finally, as the cut-point for categories for CAT severity were chosen *ad hoc *and on a purely descriptive basis rather than on empirical clinical definition, there is the possibility that where items mapped from the SGRQ-C fell close to the border between two severity categories they may have been mis-assigned. It is beyond the scope of this work to validate the CAT severity categories, and it is acknowledged that future work may be needed to prospectively both test the validity of the CAT severity categories (and SGRQ-C mapping) in a cohort of patients in whom data is collected using both SGRQ-C and the CAT, and to relate the CAT severity categories to needs of care.

An alternative approach to conveying the impact of COPD, as reflected in CAT scores, is to present a usable number of selected SGRQ items in an ascending hierarchy of severity or ladder. When using such a ladder it is important to remember that higher scores are likely to be associated with many of the milder items; a patient whose sleep is disturbed by cough or breathlessness is also likely to do housework slowly and be unable to do one or two things that they would like to do. By the same token, they are less likely to be breathless when walking around the home or have problems bathing. This COPD severity ladder is presented as an alternative approach to scenarios for providing clinicians with a picture of the life and health of a COPD patient with any given CAT score. It is important to note that it should not be used as a scale and CAT scores should not be attributed to the patient's response to selected items from this ladder - its purpose is purely illustrative.

One important contribution of this work is to focus attention on the true impact of COPD on a patient's life. In this respect, the very general adjectives used to describe the severity of the impact of the disease on the patient may be doing a disservice to the patient. A 'Medium Impact' CAT score looks anything but medium when described as a scenario, most healthy people are likely to judge that getting exhausted easily and needing to take a long time to do housework constitutes quite severe impact on health. If use of the CAT and these scenarios produces a re-evaluation of what constitutes 'mild or moderate COPD', then patients can only benefit.

## Conclusion

In conclusion, this work has shown that it is possible to relate CAT scores to scenarios descriptive of impaired health status in COPD. The CAT is a concise instrument for use in everyday clinical practice; the scenarios described here allow for a more complete understanding of what its scores reflect in terms of the effect of the disease on the patient's health. It is our hope that a more complete understanding of a COPD patient's health status may help clinicians optimise their management.

## Competing interests

**P.W.J**. has received consulting fees from Almirall, AstraZeneca, GlaxoSmithKline, Novartis, Roche and Spiration; speaking fees from AstraZeneca and GlaxoSmithKline; and grant support from GlaxoSmithKline. He received no fees or honorarium for writing this paper. **M. T**. is an employee of GlaxoSmithKline, who funded the present study and the development of the COPD Assessment Test (CAT). **W-H. C**. was employed by United BioSource Corporation at the time of the study. The present study and the development of the COPD Assessment Test (CAT) were funded by GlaxoSmithKline. COPD Assessment Test and its associated CAT logo is a trademark of the GlaxoSmithKline group of companies ^© ^2009 GlaxoSmithKline. All rights reserved.

## Authors' contributions

The authors developed the design and concept of the study, had full access to, and interpreted the resulting data, wrote the article and were responsible for decisions with regard to publication.

All authors interpreted study data, developed the first draft of the manuscript, contributed to and reviewed drafts of the manuscript, and approved the final version of the manuscript.

## Pre-publication history

The pre-publication history for this paper can be accessed here:

http://www.biomedcentral.com/1471-2466/11/42/prepub

## Supplementary Material

Additional file 1 **Appendix 1: Conversion table from CAT score to logits**. Click here for file

## References

[B1] Global Initiative for Chronic Obstructive Lung Disease (GOLD) guideline Global Strategy for the Diagnosis, management and Prevention of Chronic Obstructive Pulmonary Disease (updated 2009) http://www.goldcopd.com last accessed 12 May 2011

[B2] Cazzola M MacNee W Martinez FJ Rabe KF Franciosi LG Barnes PJ Brusasco V Burge PS Calverley PM Celli BR Jones PW Mahler DA Make B Miravitlles M Page CP Palange P Parr D Pistolesi M Rennard SI Rutten-van Mölken MP Stockley R Sullivan SD Wedzicha JA Wouters EF American Thoracic Society; European Respiratory Society Task Force on outcomes of COPD Outcomes for COPD pharmacological trials: from lung function to biomarkers Eur Respir J 2008 31 416 469 10.1183/09031936.00099306 18238951

[B3] Guyatt GH Berman LB Townsend M Pugsley SO Chambers LW A measure of quality of life for clinical trials in chronic lung disease Thorax 1987 42 773 778 10.1136/thx.42.10.773 3321537 PMC460950

[B4] van der Molen T Willemse BW Schokker S ten Hacken NH Postma DS Juniper EF Development, validity and responsiveness of the Clinical COPD Questionnaire Health Qual Life Outcomes 2003 1 13 28 10.1186/1477-7525-1-13 12773199 PMC156640

[B5] Jones PW Quirk FH Baveystock CM The St George's Respiratory Questionnaire Respir Med 1991 85 Suppl B 25 31 1759018 10.1016/s0954-6111(06)80166-6

[B6] Meguro M Barley EA Spencer S Jones PW Development and validation of an improved COPD-specific version of the St George's Respiratory Questionnaire Chest 2006 132 456 463 10.1378/chest.06-0702 17646240

[B7] Jones PW St. George's Respiratory Questionnaire: MCID COPD 2005 2 75 79 10.1081/COPD-200050513 17136966

[B8] Jones PW Interpreting thresholds for a clinically significant changes in health status in asthma and COPD Eur Respir J 2002 19 398 404 10.1183/09031936.02.00063702 11936514

[B9] Jones PW Harding G Berry P Wiklund I Chen WH Kline Leidy N Development and first validation of the COPD Assessment Test Eur Respir J 2009 34 648 654 10.1183/09031936.00102509 19720809

[B10] Jones P Harding G Wiklund I Berry P Leidy N Improving the process and outcome of care in COPD: development of a standardised assessment tool Prim Care Respir J 2009 18 208 215 10.4104/pcrj.2009.00053 19690787 PMC6619275

[B11] Bland JM Altman DG Statistical methods for assessing agreement between two methods of clinical measurement Lancet 1986 1 307 310 2868172

[B12] Rasch G Probabilistic models for some intelligence and attainment tests AA 1960 Chicago: University of Chicago Press

[B13] Barley EA Jones PW Repeatability of a Rasch model of the AQ20 over five assessments Qual Life Res 2006 15 801 809 10.1007/s11136-005-5466-z 16721640

[B14] Andrich D Sheridon BE Luo G RUMM2020 (Rasch unidimensional measurement models) AA 2005 Perth: RUMM Laboratory

[B15] Hawthorne G Densley K Pallant JF Mortimer D Segal L Deriving utility scores from the SF-36 health instrument using Rasch analysis Qual Life Res 2008 17 1183 1193 10.1007/s11136-008-9395-5 18825509

[B16] Young TA Rowen D Norquist J Brazier JE Developing preference-based health measures: using Rasch analysis to generate health state values Qual Life Res 2010 19 907 917 10.1007/s11136-010-9646-0 20454864

[B17] Wright BD Linacre JM *Glossary of Rasch Measurement Terminology *Rasch Measurement Transactions 2001 15 824 825 http://www.rasch.org/rmt/rmt152e.htm last accessed 12 May 2011

