# Supplementary material for: Creating scenarios of the impact of copd and their relationship to copd assessment test (CAT™) scores
Source: BMC Pulm Med. 2011 Aug 11;11:42. doi: 10.1186/1471-2466-11-42 (PMC3199910; doi:10.1186/1471-2466-11-42)
Supplement: Additional file 1 — Appendix 1: Conversion table from CAT score to logits. [file 1471-2466-11-42-S1.DOC]

**Appendix 1. Conversion table from CAT score to logits.**

| **CAT Score** | **CAT logits** |
| --- | --- |
| 0 | –4.31 |
| 1 | –3.45 |
| 2 | –2.87 |
| 3 | –2.46 |
| 4 | –2.16 |
| 5 | –1.90 |
| 6 | –1.69 |
| 7 | –1.50 |
| 8 | –1.33 |
| 9 | –1.18 |
| 10 | –1.03 |
| 11 | –0.90 |
| 12 | –0.78 |
| 13 | –0.66 |
| 14 | –0.54 |
| 15 | –0.43 |
| 16 | –0.33 |
| 17 | –0.23 |
| 18 | –0.13 |
| 19 | –0.03 |
| 20 | 0.07 |
| 21 | 0.16 |
| 22 | 0.26 |
| 23 | 0.35 |
| 24 | 0.45 |
| 25 | 0.55 |
| 26 | 0.64 |
| 27 | 0.75 |
| 28 | 0.85 |
| 29 | 0.96 |
| 30 | 1.07 |
| 31 | 1.19 |
| 32 | 1.32 |
| 33 | 1.47 |
| 34 | 1.63 |
| 35 | 1.82 |
| 36 | 2.04 |
| 37 | 2.31 |
| 38 | 2.67 |
| 39 | 3.21 |
| 40 | 4.01 |
